# 3-Chloro-*N*-(4-sulfamoylphen­yl)propanamide

**DOI:** 10.1107/S1600536810020465

**Published:** 2010-06-05

**Authors:** Mehmet Akkurt, Şerife Pınar Yalçın, Hasan Türkmen, Orhan Büyükgüngör

**Affiliations:** a Department of Physics, Faculty of Sciences, Erciyes University, 38039 Kayseri, Turkey; b Department of Physics, Faculty of Arts and Sciences, Harran University, 63300 Şanlıurfa, Turkey; c Department of Chemistry, Faculty of Arts and Sciences, Harran University, 63300 Şanlıurfa, Turkey; d Department of Physics, Faculty of Arts and Sciences, Ondokuz Mayıs University, 55139 Samsun, Turkey

## Abstract

In the title compound, C_9_H_11_ClN_2_O_3_S, the dihedral angle between the benzene ring and the amido –NHCO– plane is 15.0 (2)°. An intra­molecular C—H⋯O hydrogen bond generates an *S*(6) ring motif. In the crystal structure, the amino NH_2_ group is involved in inter­molecular N—H⋯O hydrogen bonds, which connect the mol­ecules into a double layer structure expanding parallel to the *bc* plane. The layers are further linked by an amido N—H⋯O hydrogen bond. Between the layers, a weak π–π inter­action with a centroid–centroid distance of 3.7447 (12) Å is also observed.

## Related literature

For the anti­bacterial and pharmacological properties of sulfonamides and their derivatives, see: Albala *et al.* (1994 ▶); Mann & Keilin (1940 ▶); Maren (1976 ▶); Pastorekova *et al.* (2004 ▶); Reynolds (1996 ▶); Silverman (1992 ▶); Supuran & Scozzafava (2001 ▶, 2002 ▶); Supuran *et al.* (2003 ▶, 2004 ▶); Türkmen *et al.* (2005 ▶). For graph-set notation, see: Bernstein *et al.* (1995 ▶).
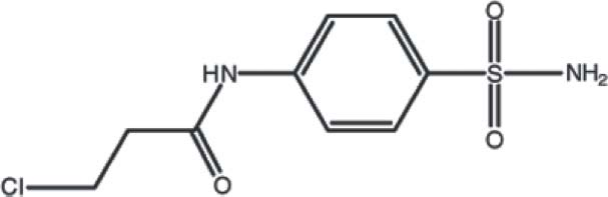


## Experimental

### 

#### Crystal data

C_9_H_11_ClN_2_O_3_S
*M* *_r_* = 262.72
Monoclinic, 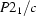

*a* = 7.7554 (4) Å
*b* = 14.8191 (8) Å
*c* = 9.7482 (5) Å
β = 94.181 (4)°
*V* = 1117.36 (10) Å^3^
*Z* = 4
Mo *K*α radiation
μ = 0.52 mm^−1^
*T* = 296 K
0.78 × 0.45 × 0.22 mm

#### Data collection

Stoe IPDS2 diffractometer
Absorption correction: integration (*X-RED32*; Stoe & Cie, 2002 ▶) *T*_min_ = 0.754, *T*_max_ = 0.892
6023 measured reflections
2294 independent reflections
2007 reflections with *I* > 2σ(*I*)
*R*_int_ = 0.040

#### Refinement

*R*[*F* ^2^ > 2σ(*F* ^2^)] = 0.038
*wR*(*F* ^2^) = 0.106
*S* = 1.08
2294 reflections
153 parameters
2 restraints
H atoms treated by a mixture of independent and constrained refinement
Δρ_max_ = 0.28 e Å^−3^
Δρ_min_ = −0.44 e Å^−3^

### 

Data collection: *X-AREA* (Stoe & Cie, 2002 ▶); cell refinement: *X-AREA* (Stoe & Cie, 2002 ▶); data reduction: *X-RED32* (Stoe & Cie, 2002 ▶); program(s) used to solve structure: *SIR97* (Altomare *et al.*, 1999 ▶); program(s) used to refine structure: *SHELXL97* (Sheldrick, 2008 ▶); molecular graphics: *ORTEP-3* (Farrugia, 1997 ▶); software used to prepare material for publication: *WinGX* (Farrugia, 1999 ▶).

## Supplementary Material

Crystal structure: contains datablocks global, I. DOI: 10.1107/S1600536810020465/is2555sup1.cif

Structure factors: contains datablocks I. DOI: 10.1107/S1600536810020465/is2555Isup2.hkl

Additional supplementary materials:  crystallographic information; 3D view; checkCIF report

## Figures and Tables

**Table 1:** Hydrogen-bond geometry (Å, °)

*D*—H⋯*A*	*D*—H	H⋯*A*	*D*⋯*A*	*D*—H⋯*A*
N1—H1*A*⋯O1^i^	0.859 (18)	2.14 (2)	2.926 (2)	151 (3)
N1—H1*B*⋯O3^ii^	0.85 (2)	2.12 (3)	2.923 (2)	158 (3)
N2—H2*A*⋯O2^iii^	0.86	2.13	2.991 (2)	175
C3—H3⋯O3	0.93	2.32	2.889 (3)	120

Symmetry codes: (i) 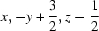; (ii) 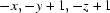; (iii) 
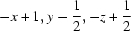
.

**Fig. 1.**
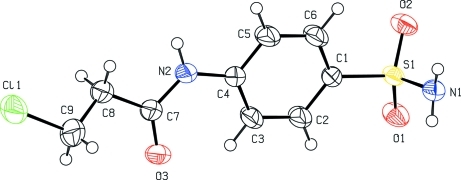
The title molecule with the atom numbering scheme. Displacement ellipsoids for non-H atoms are drawn at the 50% probability level.

**Fig. 2.**
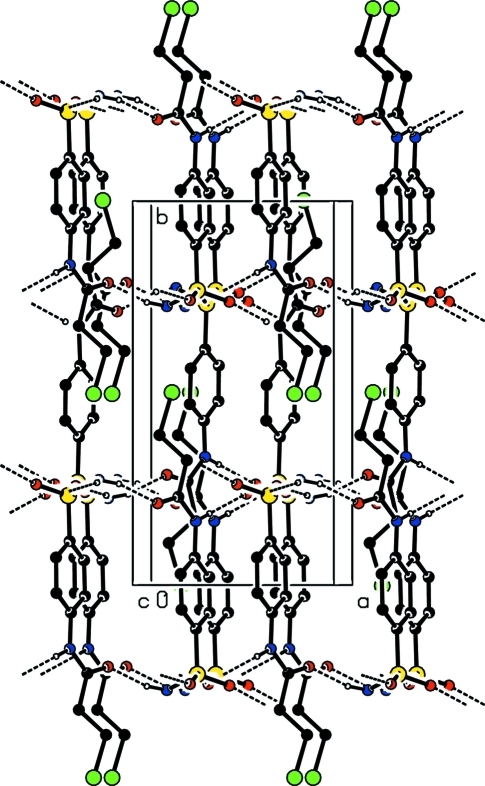
The packing of the molecules of (I) linked by of N—H···O hydrogen bonds, viewed down the c axis. All hydrogen atoms not involved in hydrogen bonding have been omitted for clarity. Hydrogen bonds are indicated by dotted lines.

## supplementary crystallographic information

### Comment

Sulfanilamide is a sulfonamide antibacterial. Chemically, it is a molecule containing the sulfonamide functional group attached to an aniline. As an antibiotic, it functions by competitively inhibiting (*i.e*, by acting as a substrate analogue) enzymatic reactions involving. Inhibition of the zinc enzyme carbonic anhydrase (CA, EC 4.2.1.1) with sulfonamides may be exploited clinically for the treatment and prevention of a multitude of diseases (Pastorekova *et al.*, 2004; Supuran *et al.*, 2004; Mann & Keilin, 1940). With the early report that sulfanilamide acts as an inhibitor of CA, a great scientific adventure initiated, leading to the development of several classes of drugs based on the sulfonamide motif.

Sulfonamides and their derivatives have been the subject of investigation for many reasons. The amides are important constituent of many biologically significant compounds. The chemistry of sulfonamides is of interest as they show distinct physical, chemical and biological properties. The sulfonamide derivatives are known for their numerous pharmacological activities, antibacterial, antitumor, insulin-release stimulation and antithyroid properties (Maren, 1976). In addition, the unsubstituted aromatic/heterocyclic sulfonamides act as carbonic anhydrase inhibitors (Supuran & Scozzafava, 2001; Türkmen *et al.*, 2005; Supuran *et al.*, 2003) whereas other types of derivatives show diuretic activity (high-ceiling diuretics or thiadiazine diuretics), hypoglycemic activity and anti- cancer properties (Supuran & Scozzafava, 2002). Although sulfonamides are best known as bacteriostatic (Silverman, 1992) and antimalarial agents (Albala *et al.*, 1994), there is now a range of drugs, possessing very different pharmacological activities, in which the sulfonamide group is present (Reynolds, 1996). Due to their significant pharmacology applications and widespread use in medicine, these compounds have gained attention in bio-inorganic and metal-based drug chemistry. In this work we report the crystal structure of 3-chloro-*N*-(4-sulfamoylphenyl)propanamide.

In the title molecule (I), (Fig. 1), the S═O distances [1.4302 (14) and 1.4349 (16) Å] and the O═S═O angle [118.21 (9)°] are within the normal range as the values of the other geometric parameters of the molecule. The dihedral angle between the benzene ring and the amido –NHCO– plane is 15.0 (2)°.

The crystal structure is stabilized by N—H···O type hydrogen bonds (Table 1, Fig. 2). N1—H1A···O1 and N1—H1B···O3 generate the two-dimensional network (double layer structure), but N2—H2A···O2 links the layers into a three-dimensional network. An intramolecular hydrogen contact C3—H3···O3 generates a ring of graph-set motif S(6) (Bernstein *et al.*, 1995) (Table 1). Furthermore, crystal packing is influenced by weak π–π stacking interactions between nearby aromatic rings of the adjacent molecules, [*Cg*···*Cg*^iv^ = 3.7447 (12) Å; *Cg* is the centroid of the C1–C6 ring; symmetry code: (iv) 1 - *x*, 1 - *y*, 1 - *z*].

### Experimental

Sulfanilamide (2.00 g, 0.011 mol) and *N*-ethylmaleimide (NEM) (1.566 g, 0.016 mol) were stirred in tetrahydrofuran (THF) (200 ml) until most of the starting material had dissolved. 3-Chloropropanoylchloride (1.782 g, 0.014 mol) in THF was slowly added to the reaction mixture. The reaction was stirred at 258 K for 4 h under anhydrous conditions. After warming to room temperature the white precipitate of NEM/HCl salt filtered off. The THF was removed in *vacuo* and the resulting white solid dissolved in ethyl acetate. The organic extract was washed with 3*M* hydrochloric acid (20 ml) then with saturated sodium bicarbonate solution (20 ml) and finally with brine. Drying over magnesium sulfate and evaporation yielded a white solid which was recrystallized from water to give the title compound (yield: 70%, m.p: 501–503 K).

### Refinement

The H-atoms of the NH_2_ group were located in a difference Fourier map, and were refined with distance restraints of N—H = 0.86 (2) Å; their temperature factors were freely refined. The other H-atoms were placed in calculated positions with C—H = 0.93–0.97 Å and N—H = 0.86 Å, and were included in the refinement in the riding model approximation, with *U*_iso_(H) = 1.2*U*_eq_(C, N).

### Crystal data

**Table:**

C_9_H_11_ClN_2_O_3_S	*F*(000) = 544
*M**_r_* = 262.72	*D*_x_ = 1.562 Mg m^−^^3^
Monoclinic, *P*2_1_/*c*	Mo *K*α radiation, λ = 0.71073 Å
Hall symbol: -P 2ybc	Cell parameters from 8775 reflections
*a* = 7.7554 (4) Å	θ = 2.1–28.0°
*b* = 14.8191 (8) Å	µ = 0.52 mm^−^^1^
*c* = 9.7482 (5) Å	*T* = 296 K
β = 94.181 (4)°	Prism, colourless
*V* = 1117.36 (10) Å^3^	0.78 × 0.45 × 0.22 mm
*Z* = 4	

### Data collection

**Table:**

Stoe IPDS2 diffractometer	2294 independent reflections
Radiation source: sealed X-ray tube, 12 x 0.4 mm long-fine focus	2007 reflections with *I* > 2σ(*I*)
plane graphite	*R*_int_ = 0.040
Detector resolution: 6.67 pixels mm^-1^	θ_max_ = 26.5°, θ_min_ = 2.5°
ω scans	*h* = −8→9
Absorption correction: integration (*X-RED32*; Stoe & Cie, 2002)	*k* = −16→18
*T*_min_ = 0.754, *T*_max_ = 0.892	*l* = −12→12
6023 measured reflections	

### Refinement

**Table:**

Refinement on *F*^2^	Primary atom site location: structure-invariant direct methods
Least-squares matrix: full	Secondary atom site location: difference Fourier map
*R*[*F*^2^ > 2σ(*F*^2^)] = 0.038	Hydrogen site location: inferred from neighbouring sites
*wR*(*F*^2^) = 0.106	H atoms treated by a mixture of independent and constrained refinement
*S* = 1.08	*w* = 1/[σ^2^(*F*_o_^2^) + (0.0602*P*)^2^ + 0.351*P*] where *P* = (*F*_o_^2^ + 2*F*_c_^2^)/3
2294 reflections	(Δ/σ)_max_ < 0.001
153 parameters	Δρ_max_ = 0.28 e Å^−^^3^
2 restraints	Δρ_min_ = −0.44 e Å^−^^3^

### Special details

**Table:**

Geometry. Bond distances, angles *etc*. have been calculated using the rounded fractional coordinates. All su's are estimated from the variances of the (full) variance-covariance matrix. The cell e.s.d.'s are taken into account in the estimation of distances, angles and torsion angles
Refinement. Refinement on *F*^2^ for ALL reflections except those flagged by the user for potential systematic errors. Weighted *R*-factors *wR* and all goodnesses of fit *S* are based on *F*^2^, conventional *R*-factors *R* are based on *F*, with *F* set to zero for negative *F*^2^. The observed criterion of *F*^2^ > σ(*F*^2^) is used only for calculating -*R*-factor-obs *etc*. and is not relevant to the choice of reflections for refinement. *R*-factors based on *F*^2^ are statistically about twice as large as those based on *F*, and *R*-factors based on ALL data will be even larger.

### Fractional atomic coordinates and isotropic or equivalent isotropic displacement parameters (Å^2^)

**Table:**

	*x*	*y*	*z*	*U*_iso_*/*U*_eq_	
Cl1	0.18392 (12)	−0.00186 (4)	0.38004 (9)	0.0791 (3)	
S1	0.31196 (6)	0.73280 (3)	0.40392 (4)	0.0330 (1)	
O1	0.2882 (2)	0.75787 (10)	0.54296 (14)	0.0471 (5)	
O2	0.4665 (2)	0.76199 (10)	0.34461 (15)	0.0445 (5)	
O3	0.1258 (2)	0.28706 (10)	0.52407 (19)	0.0571 (6)	
N1	0.1520 (2)	0.77368 (12)	0.31092 (18)	0.0398 (5)	
N2	0.3009 (2)	0.33382 (11)	0.36141 (18)	0.0419 (5)	
C1	0.3044 (2)	0.61415 (12)	0.39403 (17)	0.0331 (5)	
C2	0.2424 (3)	0.56420 (15)	0.4978 (2)	0.0499 (7)	
C3	0.2372 (4)	0.47119 (15)	0.4898 (2)	0.0530 (7)	
C4	0.2965 (2)	0.42797 (13)	0.37652 (19)	0.0360 (5)	
C5	0.3592 (3)	0.47919 (15)	0.2722 (2)	0.0508 (7)	
C6	0.3617 (3)	0.57169 (15)	0.2792 (2)	0.0488 (7)	
C7	0.2202 (3)	0.27011 (13)	0.4329 (2)	0.0389 (6)	
C8	0.2583 (3)	0.17489 (14)	0.3889 (2)	0.0448 (6)	
C9	0.1263 (4)	0.10957 (16)	0.4265 (4)	0.0725 (10)	
H1A	0.153 (4)	0.7665 (17)	0.2235 (18)	0.053 (7)*	
H1B	0.055 (3)	0.7636 (17)	0.342 (3)	0.051 (7)*	
H2	0.20340	0.59310	0.57430	0.0600*	
H2A	0.36310	0.31400	0.29840	0.0500*	
H3	0.19400	0.43770	0.56030	0.0640*	
H5	0.40020	0.45050	0.19620	0.0610*	
H6	0.40150	0.60550	0.20760	0.0590*	
H8A	0.26540	0.17370	0.29000	0.0540*	
H8B	0.36990	0.15670	0.43140	0.0540*	
H9A	0.01560	0.12520	0.38000	0.0870*	
H9B	0.11490	0.11230	0.52490	0.0870*	

### Atomic displacement parameters (Å^2^)

**Table:**

	*U*^11^	*U*^22^	*U*^33^	*U*^12^	*U*^13^	*U*^23^
Cl1	0.1120 (6)	0.0337 (3)	0.0982 (6)	−0.0094 (3)	0.0520 (5)	−0.0065 (3)
S1	0.0424 (3)	0.0302 (2)	0.0276 (2)	−0.0060 (2)	0.0106 (2)	−0.0011 (2)
O1	0.0702 (10)	0.0434 (8)	0.0294 (7)	−0.0105 (7)	0.0146 (6)	−0.0057 (5)
O2	0.0459 (8)	0.0452 (8)	0.0440 (8)	−0.0140 (6)	0.0142 (6)	0.0000 (6)
O3	0.0628 (10)	0.0383 (8)	0.0757 (11)	−0.0035 (7)	0.0427 (9)	−0.0018 (7)
N1	0.0471 (10)	0.0372 (9)	0.0367 (9)	0.0024 (7)	0.0131 (7)	0.0019 (7)
N2	0.0504 (10)	0.0315 (8)	0.0466 (9)	0.0014 (7)	0.0233 (7)	−0.0011 (7)
C1	0.0382 (10)	0.0295 (9)	0.0323 (8)	0.0002 (7)	0.0075 (7)	0.0012 (6)
C2	0.0740 (15)	0.0352 (10)	0.0443 (11)	0.0005 (10)	0.0310 (10)	0.0001 (8)
C3	0.0814 (16)	0.0348 (11)	0.0473 (11)	0.0008 (11)	0.0354 (11)	0.0060 (9)
C4	0.0383 (10)	0.0315 (9)	0.0394 (9)	0.0021 (8)	0.0116 (8)	0.0022 (7)
C5	0.0732 (15)	0.0387 (10)	0.0447 (11)	−0.0035 (10)	0.0323 (11)	−0.0041 (9)
C6	0.0712 (15)	0.0377 (10)	0.0410 (10)	−0.0061 (10)	0.0278 (10)	0.0009 (8)
C7	0.0385 (10)	0.0335 (10)	0.0461 (11)	−0.0008 (8)	0.0126 (8)	0.0004 (8)
C8	0.0500 (12)	0.0344 (10)	0.0520 (11)	−0.0010 (9)	0.0176 (9)	−0.0027 (8)
C9	0.0714 (18)	0.0328 (11)	0.118 (2)	−0.0020 (12)	0.0382 (17)	−0.0012 (13)

### Geometric parameters (Å, °)

**Table:**

Cl1—C9	1.778 (3)	C3—C4	1.384 (3)
S1—O1	1.4302 (14)	C4—C5	1.385 (3)
S1—O2	1.4349 (16)	C5—C6	1.373 (3)
S1—N1	1.6012 (17)	C7—C8	1.510 (3)
S1—C1	1.7617 (18)	C8—C9	1.475 (4)
O3—C7	1.218 (3)	C2—H2	0.9300
N2—C4	1.404 (3)	C3—H3	0.9300
N2—C7	1.354 (3)	C5—H5	0.9300
N1—H1A	0.859 (18)	C6—H6	0.9300
N1—H1B	0.85 (2)	C8—H8A	0.9700
N2—H2A	0.8600	C8—H8B	0.9700
C1—C2	1.369 (3)	C9—H9A	0.9700
C1—C6	1.385 (3)	C9—H9B	0.9700
C2—C3	1.381 (3)		
			
Cl1···N1^i^	3.3993 (19)	C3···O3	2.889 (3)
Cl1···C9^ii^	3.543 (3)	C7···O2^vi^	3.173 (3)
Cl1···H9B^ii^	3.0400	C8···O2^vi^	3.372 (3)
S1···O1^iii^	3.5128 (14)	C9···Cl1^ii^	3.543 (3)
O1···N1^iv^	2.926 (2)	C7···H3	2.7900
O1···S1^iv^	3.5128 (14)	C8···H6^x^	3.0400
O1···O2^iv^	3.171 (2)	H1A···O1^iii^	2.14 (2)
O2···N2^v^	2.992 (2)	H1A···H2^iii^	2.5800
O2···C8^vi^	3.372 (3)	H1B···O3^vii^	2.12 (3)
O2···O1^iii^	3.171 (2)	H2···O1	2.5500
O2···C7^vi^	3.173 (3)	H2···H1A^iv^	2.5800
O3···N1^vii^	2.923 (2)	H2A···H5	2.2800
O3···C3	2.889 (3)	H2A···H8A	2.2100
O1···H6^iv^	2.6900	H2A···O2^x^	2.1300
O1···H1A^iv^	2.14 (2)	H3···O3	2.3200
O1···H2	2.5500	H3···C7	2.7900
O2···H8A^v^	2.8600	H5···H2A	2.2800
O2···H6	2.7100	H6···O2	2.7100
O2···H2A^v^	2.1300	H6···C8^v^	3.0400
O2···H8B^vi^	2.7200	H6···H8B^v^	2.4300
O3···H9A	2.8800	H6···O1^iii^	2.6900
O3···H9B	2.5900	H8A···H2A	2.2100
O3···H3	2.3200	H8A···O2^x^	2.8600
O3···H1B^vii^	2.12 (3)	H8A···O3^xi^	2.8000
O3···H8A^viii^	2.8000	H8B···H6^x^	2.4300
N1···Cl1^ix^	3.3993 (19)	H8B···O2^vi^	2.7200
N1···O3^vii^	2.923 (2)	H9A···O3	2.8800
N1···O1^iii^	2.926 (2)	H9B···O3	2.5900
N2···O2^x^	2.991 (2)	H9B···Cl1^ii^	3.0400
			
O1—S1—O2	118.21 (9)	N2—C7—C8	113.46 (18)
O1—S1—N1	106.87 (9)	O3—C7—C8	122.71 (18)
O1—S1—C1	107.76 (8)	C7—C8—C9	112.9 (2)
O2—S1—N1	107.05 (9)	Cl1—C9—C8	110.8 (2)
O2—S1—C1	107.72 (8)	C1—C2—H2	120.00
N1—S1—C1	108.98 (9)	C3—C2—H2	120.00
C4—N2—C7	128.58 (17)	C2—C3—H3	120.00
S1—N1—H1A	117 (2)	C4—C3—H3	120.00
S1—N1—H1B	113.8 (19)	C4—C5—H5	120.00
H1A—N1—H1B	114 (3)	C6—C5—H5	119.00
C4—N2—H2A	116.00	C1—C6—H6	120.00
C7—N2—H2A	116.00	C5—C6—H6	120.00
S1—C1—C2	120.76 (14)	C7—C8—H8A	109.00
S1—C1—C6	119.09 (14)	C7—C8—H8B	109.00
C2—C1—C6	120.15 (18)	C9—C8—H8A	109.00
C1—C2—C3	120.56 (19)	C9—C8—H8B	109.00
C2—C3—C4	119.8 (2)	H8A—C8—H8B	108.00
N2—C4—C5	117.07 (17)	Cl1—C9—H9A	109.00
C3—C4—C5	119.15 (19)	Cl1—C9—H9B	109.00
N2—C4—C3	123.77 (18)	C8—C9—H9A	110.00
C4—C5—C6	121.01 (19)	C8—C9—H9B	109.00
C1—C6—C5	119.32 (19)	H9A—C9—H9B	108.00
O3—C7—N2	123.84 (18)		
			
O1—S1—C1—C2	14.47 (18)	C2—C1—C6—C5	−1.5 (3)
O2—S1—C1—C2	143.03 (16)	C6—C1—C2—C3	0.4 (3)
N1—S1—C1—C2	−101.16 (17)	C1—C2—C3—C4	0.6 (4)
O1—S1—C1—C6	−165.74 (15)	C2—C3—C4—N2	178.1 (2)
O2—S1—C1—C6	−37.18 (17)	C2—C3—C4—C5	−0.5 (3)
N1—S1—C1—C6	78.63 (17)	N2—C4—C5—C6	−179.24 (19)
C7—N2—C4—C3	15.1 (3)	C3—C4—C5—C6	−0.6 (3)
C7—N2—C4—C5	−166.4 (2)	C4—C5—C6—C1	1.6 (3)
C4—N2—C7—O3	1.0 (3)	O3—C7—C8—C9	21.5 (3)
C4—N2—C7—C8	−178.99 (18)	N2—C7—C8—C9	−158.5 (2)
S1—C1—C2—C3	−179.80 (19)	C7—C8—C9—Cl1	−177.00 (18)
S1—C1—C6—C5	178.75 (17)		

Symmetry codes: (i) *x*, *y*−1, *z*; (ii) −*x*, −*y*, −*z*+1; (iii) *x*, −*y*+3/2, *z*−1/2; (iv) *x*, −*y*+3/2, *z*+1/2; (v) −*x*+1, *y*+1/2, −*z*+1/2; (vi) −*x*+1, −*y*+1, −*z*+1; (vii) −*x*, −*y*+1, −*z*+1; (viii) *x*, −*y*+1/2, *z*+1/2; (ix) *x*, *y*+1, *z*; (x) −*x*+1, *y*−1/2, −*z*+1/2; (xi) *x*, −*y*+1/2, *z*−1/2.

### Hydrogen-bond geometry (Å, °)

**Table:**

*D*—H···*A*	*D*—H	H···*A*	*D*···*A*	*D*—H···*A*
N1—H1A···O1^iii^	0.859 (18)	2.14 (2)	2.926 (2)	151 (3)
N1—H1B···O3^vii^	0.85 (2)	2.12 (3)	2.923 (2)	158 (3)
N2—H2A···O2^x^	0.86	2.13	2.991 (2)	175
C3—H3···O3	0.93	2.32	2.889 (3)	120

Symmetry codes: (iii) *x*, −*y*+3/2, *z*−1/2; (vii) −*x*, −*y*+1, −*z*+1; (x) −*x*+1, *y*−1/2, −*z*+1/2.

## References

[bb1] Albala, D. M., Prien, E. L. & Galal, H. A. (1994). *J. Endourol.* **8**, 401–403. 10.1089/end.1994.8.401 7703990

[bb2] Altomare, A., Burla, M. C., Camalli, M., Cascarano, G. L., Giacovazzo, C., Guagliardi, A., Moliterni, A. G. G., Polidori, G. & Spagna, R. (1999). *J. Appl. Cryst.* **32**, 115–119.

[bb3] Bernstein, J., Davis, R. E., Shimoni, L. & Chang, N.-L. (1995). *Angew. Chem. Int. Ed. Engl.* **34**, 1555–1573.

[bb4] Farrugia, L. J. (1997). *J. Appl. Cryst.* **30**, 565.

[bb5] Farrugia, L. J. (1999). *J. Appl. Cryst.* **32**, 837–838.

[bb6] Mann, T. & Keilin, D. (1940). *Nature* (London), **164**, 146–148.

[bb7] Maren, T. H. (1976). *Annu. Rev. Pharmacol. Toxicol.* **16**, 309–327. 10.1146/annurev.pa.16.040176.001521 59572

[bb8] Pastorekova, S., Parkkila, S., Pastorek, J. & Supuran, C. T. J. (2004). *J. Enzyme Inhib. Med. Chem.* **19**, 199–229. 10.1080/14756360410001689540 15499993

[bb9] Reynolds, J. E. F. (1996). Editor. *Martindale: The Extra Pharmacopoeia*, 31st ed. London: The Royal Pharmaceutical Society.

[bb10] Sheldrick, G. M. (2008). *Acta Cryst.* A**64**, 112–122. 10.1107/S0108767307043930 18156677

[bb11] Silverman, R. B. (1992). *The Organic Chemistry of Drug Design and Drug Action* London: Academic.

[bb12] Stoe & Cie (2002). *X-AREA* and *X-RED32* Stoe & Cie, Darmstadt, Germany.

[bb13] Supuran, C. T. & Scozzafava, A. (2001). *Curr. Med. Chem. Immunol. Endocrinol. Metab. Agent.* **1**, 61–97.

[bb14] Supuran, C. T. & Scozzafava, A. (2002). *Expert Opin. Ther. Patents*, **12**, 217–242.

[bb15] Supuran, C. T., Scozzafava, A. & Casini, A. (2003). *Med. Res. Rev.* **23**, 146–189. 10.1002/med.10025 12500287

[bb16] Supuran, C. T., Vullo, D., Manole, G., Casini, A. & Scozzafava, A. (2004). *Curr. Med. Chem. Cardiovasc. Hematol. Agents*, **2**, 49–68. 15328829

[bb17] Türkmen, H., Durgun, M., Yılmaztekin, S., Emul, M., Innocenti, A., Vullo, D., Scozzafava, A. & Supuran, C. T. (2005). *Bioorg. Med. Chem. Lett.* **15**, 367–372. 10.1016/j.bmcl.2004.10.070 15603956

